# Impact of acute stress on the canine gut microbiota

**DOI:** 10.1038/s41598-024-66652-3

**Published:** 2024-08-14

**Authors:** Krusha V. Patel, Alysia B. G. Hunt, Juan Castillo-Fernandez, Christine Abrams, Tammie King, Phillip Watson, Gregory C. A. Amos

**Affiliations:** Waltham Petcare Science Institute, Freeby Lane, Waltham On the Wolds, Leicestershire, LE14 4RT UK

**Keywords:** Dog, Microbiota, Faeces, Stress, Car travel, Separation, Microbiology, Stress and resilience

## Abstract

There is growing evidence that a relationship exists between mental and emotional wellbeing and the gut microbiota. Little is known regarding how the microbiota reacts to repeated acute stress events in dogs, and whether it is a predictor of stress response. In this study, we explored the impact on the gut microbiota and digestive health with two common events many pet dogs find stressful. Twenty healthy adult dogs, living within a colony, were exposed to either car travel or separation three times across eight-week intervals. Faecal samples were collected 24 h before, within 24 h, and 24–48 h after. Faecal quality and pH, and microbiota diversity and composition were analysed in context with wider published work on physiological stress measures. No significant changes were observed in faecal quality or pH with either stress event at any timepoint, indicating all pets remained in good digestive health. Microbiota analysis demonstrated no significant impact on alpha or beta diversity with either stressor. Microbial signatures previously linked to stress were not identified in these dogs and no changes were observed in the functional gut composition. Irrespective of whether the pet was considered “stressed” (i.e., exhibited an increase in serum cortisol), there was no effect on the microbiota and no taxa were predictive of stress response. Collectively, this work demonstrates, for this population, certain acute stress events have no meaningful impact on the canine gut microbiota, and it has no impact on the associated stress response.

## Introduction

The gastrointestinal microbiome is increasingly understood to play a vital role in the overall health and nutritional status of mammals, with a growing interest in its relationship with the central nervous system (CNS)^1,2^. The gut microbiome influences host behaviour via the CNS through several channels, and conversely the CNS can modulate its environment^3,4^. Most research suggests behaviour is impacted through vagal nerve neurons detecting changes in metabolites produced by the gut microbiota, such as neurotransmitters or other molecules, including γ-aminobutyric acid (GABA), amino-acid derivatives such as serotonin, or catecholamines such as dopamine and norepinephrine^5^. The gut-brain axis in mammalian species has been discussed in a review exploring the use of canine in vitro and in vivo disease models as surrogates to further the understanding of therapeutics for severe neurodegenerative disorders such as Parkinson’s and Alzheimer’s Disease in humans^6^. Studies in mice have demonstrated that even a short-term exposure to stress, and as little as two hours, can impact the microbiota^7^. It has been suggested that dysbiosis, or a disruption of the gut microbiota, in humans could be involved in the pathophysiology of stress-related psychiatric disorders, including depression^8^.

Changes in neurological states, such as stress, could inhibit a vagal tone, resulting in shifts in the microbial community, with mice model studies demonstrating a strong link between the microbiota and anxiety-like behaviour^9,10^. As such, exposure to stress may affect the microbial composition through reducing health-associated bacterial species and proliferating those that are possibly pathogenic^11^. A recent review exploring the gut-brain axis across humans, rodents, and dogs highlighted the scarcity of publications in canines with the specific link to anxiety and/or stress, and in turn the limitations of understanding^12^. One study exploring the gut microbiota of anxious dogs (as classified by at least one of the following: “always alert”, “difficulty relaxing”, “fearful of noises, people, animals”, and “aggressive behaviours”, among others) showed increased *Lactobacillus* sp., *Bifidobacteria*, and *Enterobacteriaceae* compared to healthy counterparts^13^. Another canine study reported significant differences in the gut microbiota of aggressive and non-aggressive dogs; an increased abundance of *Lactobacillaceae* was found in aggressive dogs, whilst *Fusobacteriaceae* were more abundant in non-aggressive dogs^14^.

Certain real-world situations are likely to elicit stress and anxiety in dogs, such as car travel due to the combination of motion, noise, and vibrations^15,16^. Car travel stress can manifest in a variety of ways in dogs, including whining, barking, and vomiting^17,18^. These behaviours can negatively impact both dog and human welfare. Another common stress event many pet dogs experience is being socially isolated from caregivers, which can result in separation-related anxiety. In a global study conducted with dog owners to understand the prevalence of canine behaviour issues, separation-related anxiety appeared in 13% of the reports^19^. Furthermore, separation-related problem behaviours in dogs are one of the most reported issues to pet behaviourists^20^. Extreme symptomatic behaviours such as elimination, barking, and destruction, that are understandably harmful to the human-animal bond, are often cited^21^. It is not uncommon for pet dogs to be left alone for long periods, with some individuals better able to cope than others. However, there is value in understanding what impact being left alone for shorter periods of time has on dogs as pet owners may be wrongly assuming that their pet is coping with brief periods of social isolation. This is especially likely if the dog does not exhibit outward signs of distress and there are no obvious problematic concerns to the caregiver. These commonly occurring events, which many pet dogs are exposed to daily, may result in negative experiences which can have long-lasting effects on individuals, impacting their immunity, general health, and behaviour^22^.

As such, we sought to understand the influence of these types of events, that may be considered stressful to pet dogs, on the gut microbiota. Furthermore, we wanted to understand whether the canine microbiota could predict the stress response to these events, alongside the measurement of serum cortisol as an indicator of stress. To do this, we performed analysis of the gut microbiota and faecal quality measures on pets undergoing simulated real-world events including being isolated from caregivers, as well as travelling in a vehicle. We conducted a controlled study exposing a healthy, mixed-breed cohort of adult colony dogs to an acute stress paradigm to mimic events experienced by pet dogs in real life. The events were repeated such that three exposures took place with eight-week intervals between each. To determine the impact of stress, we compared the gut microbiota both before and after the stress events and used previously published data on the effects of serum cortisol to understand whether there were taxa that could predict a stress response in dogs^23^.

## Results

### Canine faecal quality score and faecal pH are not influenced by acute stress events

**Figure 1:**
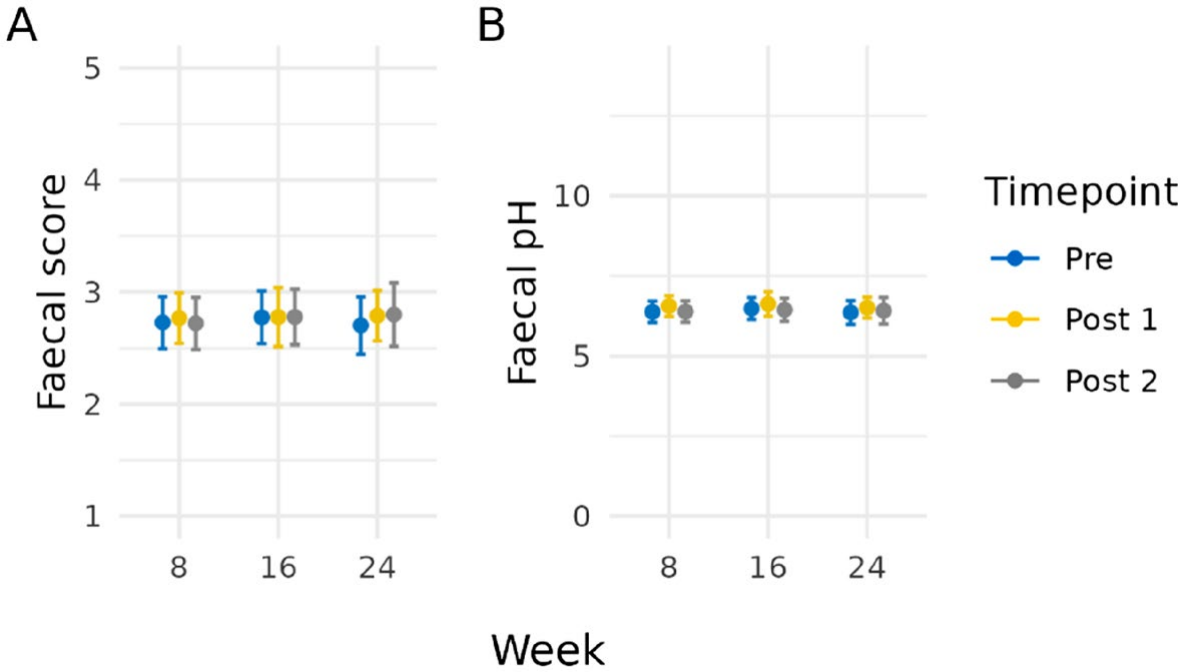
**(A)** Faecal score mean values for all dogs across three timepoints (Weeks 8, 16, and 24). **(B)** Faecal pH mean values for all dogs across three timepoints (Weeks 8, 16, and 24). The sample collection times have been represented as Pre (24 h prior to the stress event) = blue, Post 1 (within 24 h of the stress event) = yellow, and Post 2 (between 24 to 48 h after the stress event) = grey. Error bars represent 95% confidence intervals.

To determine whether there was an influence on digestive health with acute stress, we explored the faecal quality score before and after two distinct paradigms: car travel and separation. These were tested three times at eight-week intervals (Fig. 1A). To explore the faecal scores before inducing stress in dogs, a faecal sample was collected 24 h prior to the stress event (Pre); two samples were collected after the event, i.e., Post 1 (within 24 h) and Post 2 (between 24 to 48 h). Across all dogs, both before and after the acute stress events, variation in faeces scores were observed. However, all nine timepoints still fell within the range of acceptable faeces, with means ranging from 2.70 to 2.80^24^. This suggests there is no impact on faecal quality with the stressors from either a car journey or separation.

We next explored whether pH-related changes could be detected in the collected faecal samples owing to faecal pH being a strong indicator of pathogen colonisation resistance^25^. Comparing the faecal pH across timepoints showed an apparent trend towards an increase in the mean of the Post 1 samples, however this was not significantly different (Fig. 1B). For Week 8, this mean increase from the Pre sample to Post 1 was 0.18 (95% CI (− 0.19, 0.55)) with a *p*-value of 0.72; Week 16 was 0.15 (95% CI (− 0.28, 0.57)), *p*-value of 0.92; Week 24 was 0.15 (95% CI (− 0.25, 0.55)), *p*-value of 0.89.

### Bacterial diversity is not influenced by acute stress events

Stress has been proposed as an environmental factor that can induce microbiome dysbiosis. To explore the influence of stress on the gut microbiota of canines, faecal samples collected from before and after exposure to the stress paradigms were explored by shallow shotgun sequencing. From the twenty healthy adult dogs, a total of 135 faeces samples were profiled, resulting in the identification of 557 taxa at species level or lowest common ancestor when mapping ties were identified.

**Figure 2:**
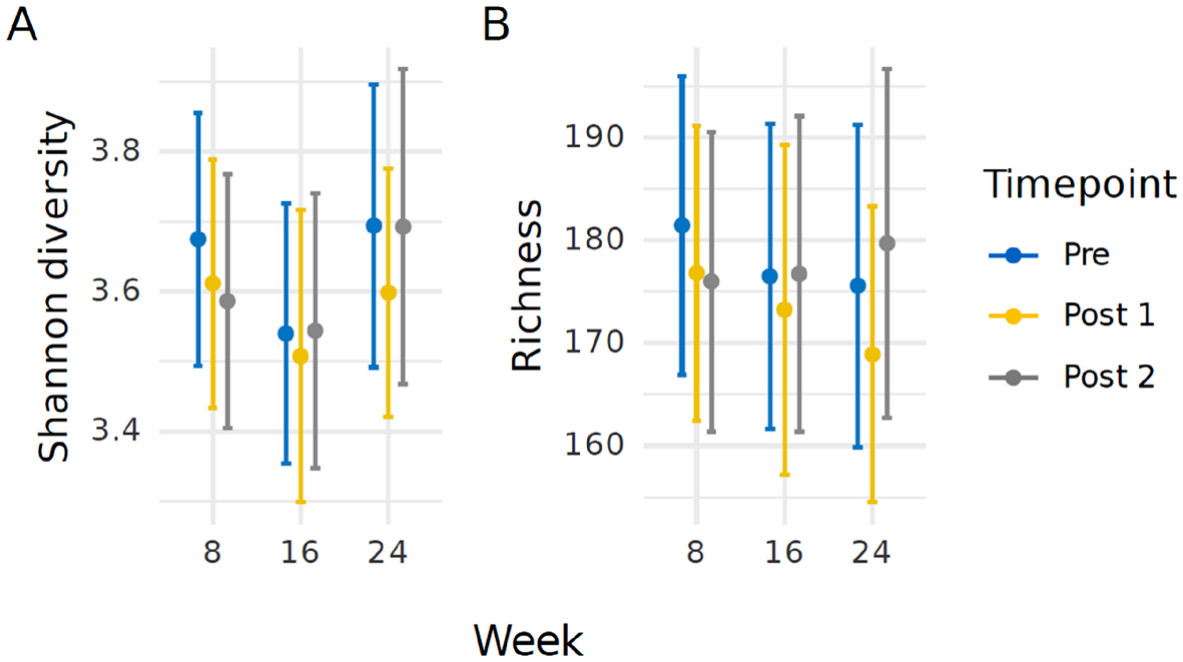
**(A)** Shannon diversity index mean values for all dogs across three timepoints (Weeks 8, 16, and 24). **(B)** Species richness mean values for all dogs across three timepoints (Weeks 8, 16, and 24). The sample collection times have been represented as Pre (24 h prior to the stress event) = blue, Post 1 (within 24 h of the stress event) = yellow, and Post 2 (between 24 to 48 h after the stress event) = grey. Error bars represent 95% confidence intervals.

Bacterial alpha diversity was estimated using Shannon diversity index scores, accounting for both abundance and evenness of the species present within a sample (Fig. 2A). These were assessed within 24 h prior to exposure to one of the stress-inducing paradigms, and twice after (Post 1: within 24 h and Post 2: within 48 h). Dogs were exposed to each stress event on three separate occasions. As such, the mean diversity has been calculated across these nine timepoints. No significant changes were observed across all samples and all comparisons that we conducted.

For Week 8, the mean diversity was 3.67 (95% CI (3.49, 3.85)); although this fell to 3.61 (95% CI (3.43, 3.79)) after the dogs experienced the stress event for the Post 1 sample and decreased again to 3.58 (95% CI (3.40, 3.77)) for the Post 2 timepoint. However, pair-wise comparisons determined these changes as not statistically significant. For Week 24, there was a decrease between Pre and Post 1 from 3.69 (95% CI (3.49, 3.90)) to 3.60 (95% CI (3.42, 3.77)); these were also not statistically significant. Comparisons between Pre to Post 1 and Pre to Post 2 were conducted, however no significant findings were recorded. Finally, comparisons were explored between Pre samples obtained from Weeks 8, 16, and 24 to elucidate changes in diversity across repeated exposures to stress. Again, no significant changes were found.

Species richness was also explored (Fig. 2B). As before, comparisons were made across different timepoints and across weeks. As with the Shannon diversity, an overall stability in the means was observed across the nine stress exposures, with a range between 167.77 to 180.97. The largest difference was again observed at Week 24, where the Pre sample mean was 175.36 (95% CI (159.66, 191.06)), decreased to 167.77 (95% CI (153.33, 182.21)), before increasing to 179.32 (95% CI (162.36, 196.29)). Pair-wise comparisons for these and all other timepoints again resulted in no statistical significance.

For both Shannon diversity and species richness, the mean differences were also explored further based on the type of stress event, i.e., car travel or separation. This did not affect the overall results observed, nor the lack of associated significance. However, it is worth noting that by sub-classifying the datasets into groups, this will have limited the analysis power.

### Beta diversity is not influenced by acute stress events

**Figure 3:**
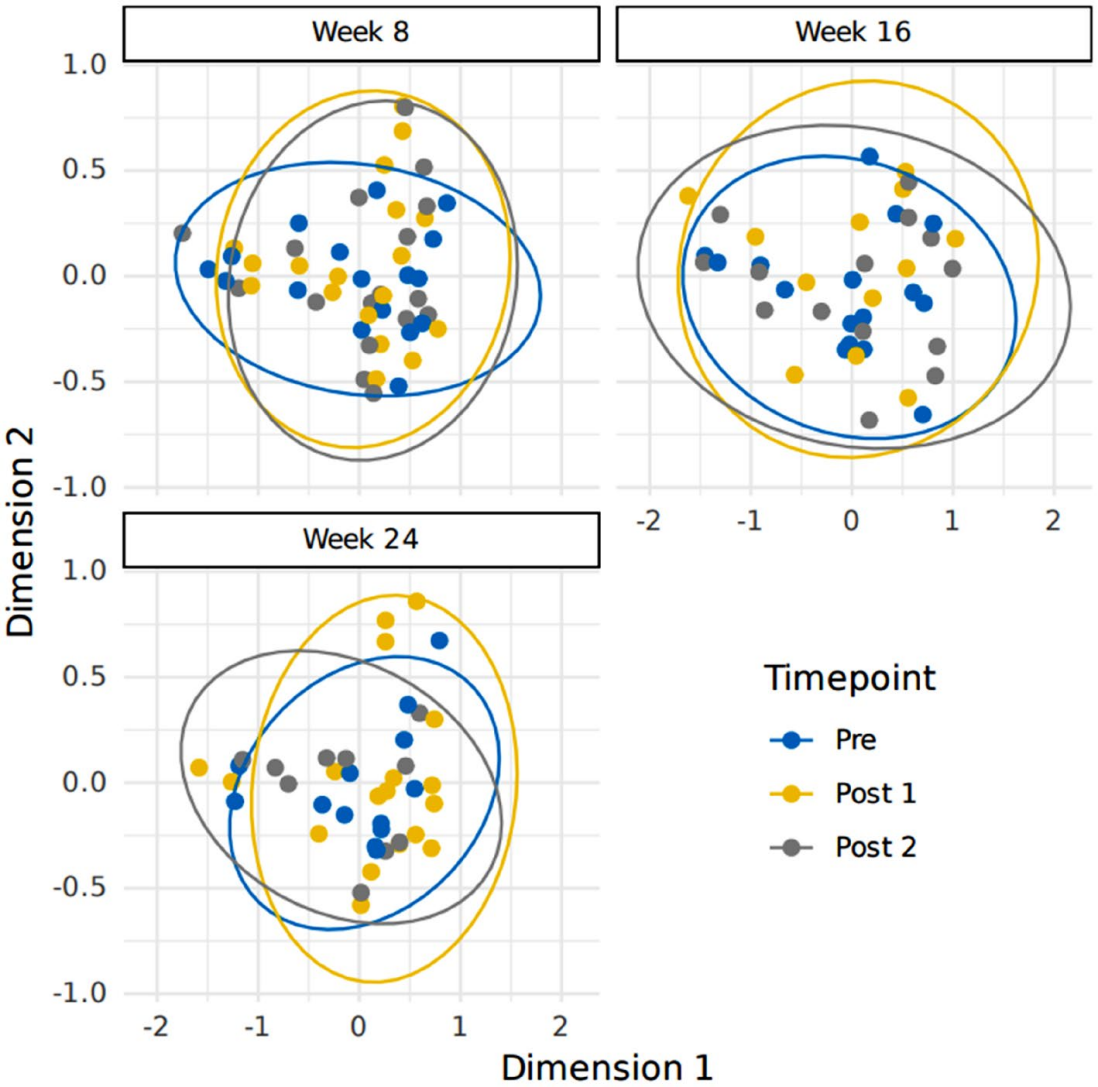
Bray-Curtis (Non-metric Multi-dimensional Scaling; nMDS) taxonomic beta diversity plot to explore sample dissimilarity across three timepoints (Weeks 8, 16, and 24). The sample collection times have been represented as Pre (24 h prior to the stress event) = blue, Post 1 (within 24 h of the stress event) = yellow, and Post 2 (between 24 to 48 h after the stress event) = grey. Ellipses represent 95% confidence intervals.

Taxonomic beta diversity was calculated with Bray-Curtis, and nMDS used to visually assess dissimilarities. This was explored across the different weeks, and across all timepoints (Fig. 3). This showed no distinction from the samples collected before stress event (Pre) to those from either sample after the exposure (Post 1 or Post 2) from any of the study weeks. There were also no differences observed between the Pre samples for Weeks 8 vs. 16 vs. 24. Overlapping ellipses showed strong similarity with the communities. These observations concurred with pair-wise comparisons with PERMANOVA and Bonferroni adjustments. For example, when comparing the Pre sample from Week 8 to the associated Post 1 and Post 2 samples, R^2^ values of 0.01 (adjusted *p*-value = 1.00) and 0.02 (adjusted *p*-value = 1.00) were obtained, respectively. We then performed PERMANOVA on KEGG KOs and observed no significant functional beta diversity clustering between timepoints at any of the different weeks (Suppl. Table 1).

**Figure 4:**
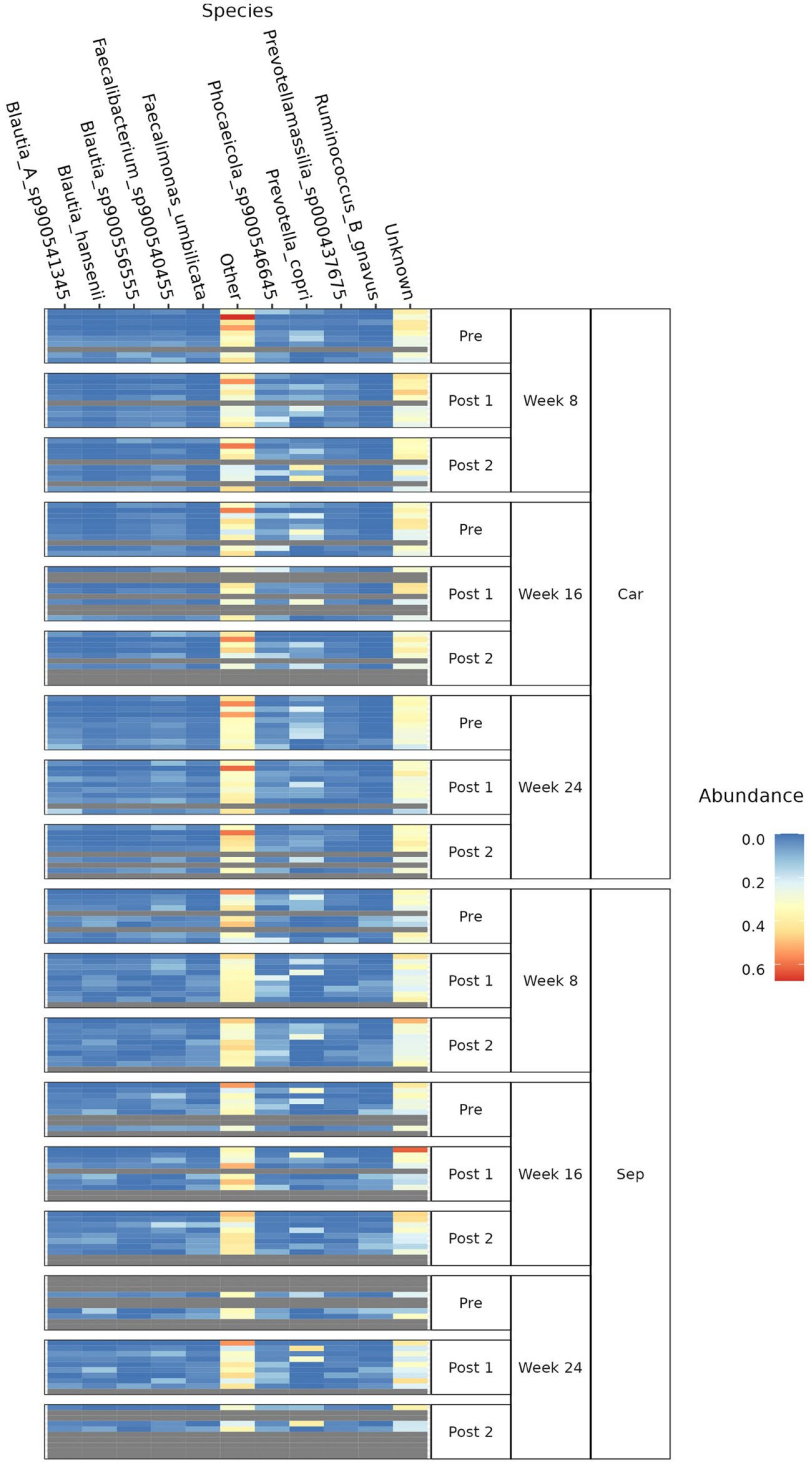
Heatmap of the nine most abundant species identified across three timepoints (Weeks 8, 16, and 24) and by stress paradigm: car travel (Car) and separation (Sep). Unknown = species that could not be assigned taxonomy; Other = species that could be assigned taxonomy but were less abundant than the top nine. Sample collection times have been represented as Pre (24 h prior to the stress event), Post 1 (within 24 h of the stress event), and Post 2 (between 24 to 48 h after the stress event). Abundance is represented with the change in colour from blue to red. Each line is a canine faecal sample; dog sample order is repeated across all nine timepoints, with grey lines indicating missing samples.

Across the most abundant species, there were no significant differences between animals who were exposed to car travel across the nine timepoints or against individual test weeks. This was also the case for dogs exposed to the separation event (Fig. 4; Suppl. Table 2 and Suppl. Table 3). However, it was observed that regardless of which stress paradigm or timepoint explored, the most abundant bacteria were various *Blautia* species, *Faecalibacterium* sp., *Prevotella* sp., and *Ruminococcus B gnavus*.

### Stress response does not correlate with the pre-existing canine gut microbiota

**Figure 5:**
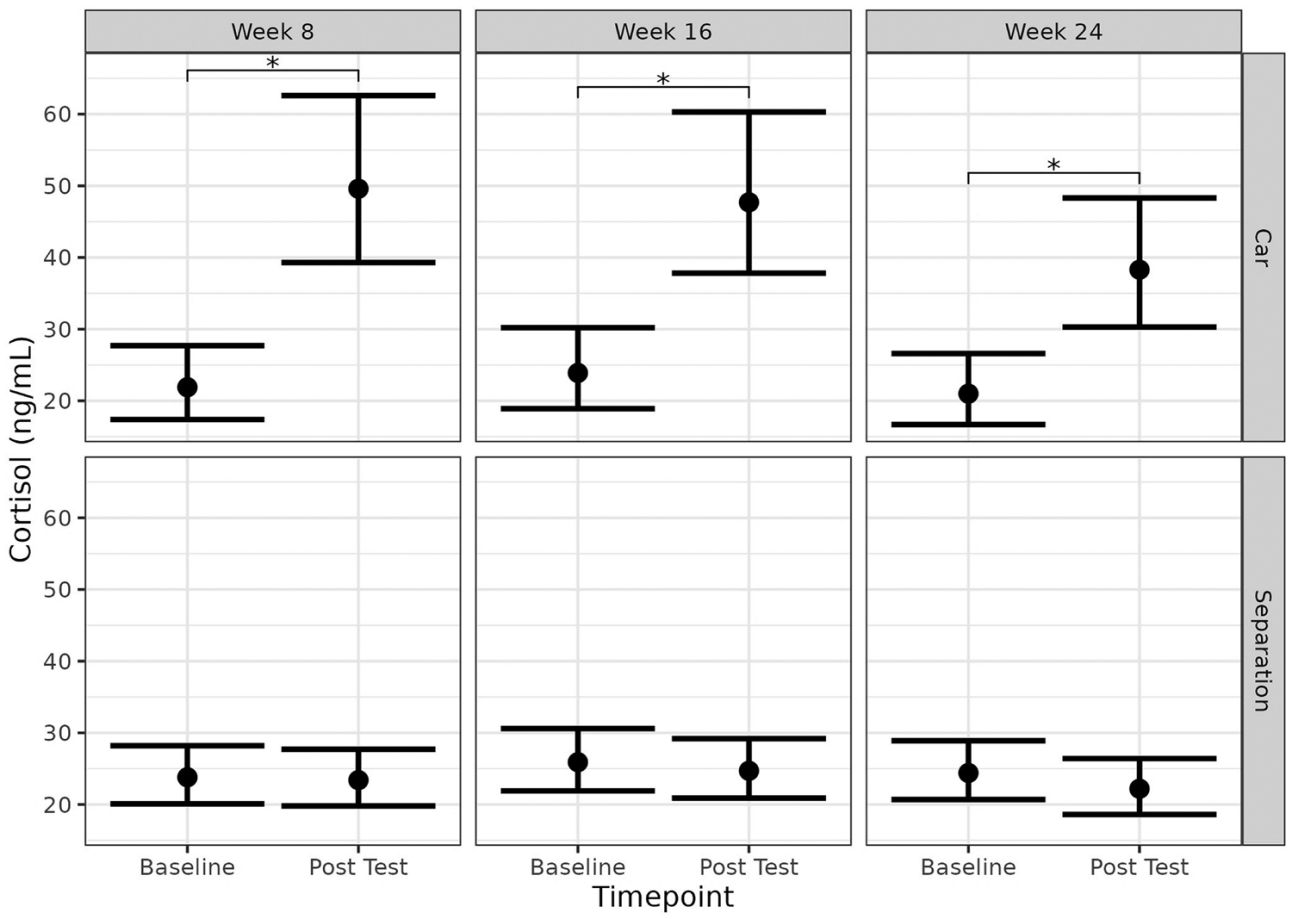
Serum cortisol (ng/mL) concentrations for dogs prior to and after exposure to stress paradigm (“Baseline” and “Post Test”, respectively). Plots have been created to represent each timepoint (Weeks 8, 16, and 24) and the associated stress paradigm (“Car” or “Separation”). Asterisks indicate *p*-values below 0.05 and significant differences between treatment groups within each phase. Error bars represent 95% confidence intervals. Reproduced using data from Hunt et al. (2023)^23^.

Serum cortisol samples were taken at Baseline (i.e., before exposure to the stress test) and Post Test (i.e., immediately after exposure to the stress test) across Weeks 8, 16, and 24, as described in a previously published parallel study^23^ (Fig. 5). Using this data, we determined there was a significant difference in detected levels of cortisol from the dogs who underwent car travel. As such, this stress paradigm was successful in inducing stress in this specific population of dogs. However, when observing the impact that the separation event had, there were no significant differences observed. This justifies analysing the microbiota data separately, albeit with the caveat of reducing the sample size. This also concurred with the findings from the group when reconciling all the behavioural and physiological markers into a “combined stress score”^23^.

**Figure 6:**
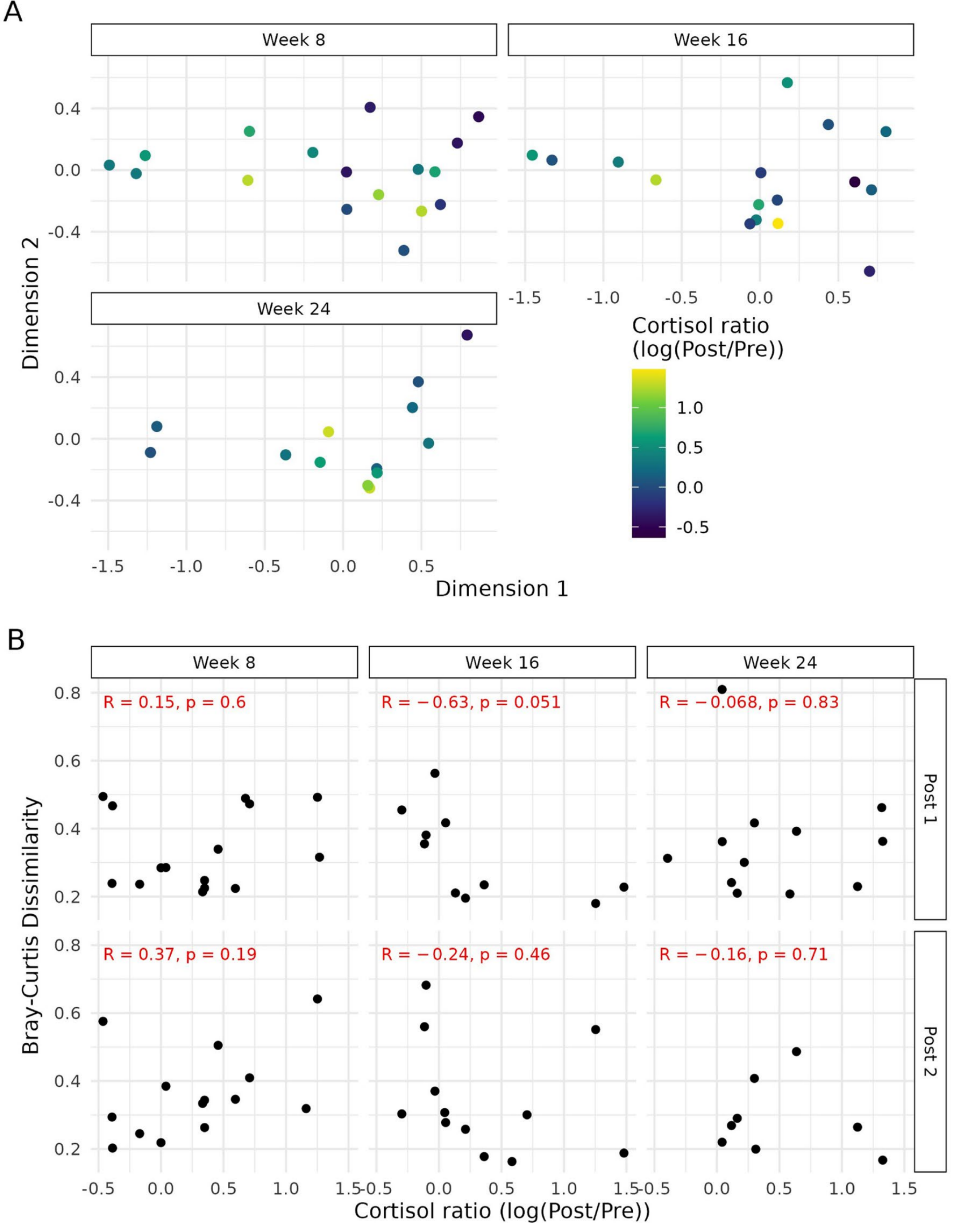
**(A)** Bray-Curtis (nMDS) beta diversity plot to represent the log difference obtained in serum cortisol (ng/mL) across three timepoints (Weeks 8, 16, and 24). Blue colour scale used with a darker shade indicative of a greater observed difference. **(B)** Scatter plot of Bray-Curtis dissimilarity when comparing either Post 1 or Post 2 samples to their respective Pre sample vs. the log difference obtained in serum cortisol (ng/mL). *P*-values below 0.05 were considered statistically significant.

To determine whether the baseline microbiota (pre-exposure) has a relationship with the cortisol response (difference from the Baseline and Post Test serum cortisol levels), we used cortisol data generated from a parallel study with the same dog cohort and correlated it with microbiota composition (Fig. 6A,B). No relationship was observed in any of the three test weeks. Then, to determine whether a change in cortisol had a relationship with a change in the microbial community, we correlated the Bray-Curtis dissimilarity between the Pre and Post samples of the same dog and its corresponding change in cortisol. The changes in serum cortisol levels were visualised with the Post 1 and Post 2 faecal samples separated. Across (Week 8, 16, and 24) and within (Post 1 and Post 2) timepoints, there was no consistency observed with the *R*-values. The *R-*value is a correlation coefficient that represents how closely two variables are related. No trends could be observed when exploring the Pre to Post 1 and Pre to Post 2 serum cortisol differences. Furthermore, the *p*-values indicate the lack of significance. As such, comparing the microbiota present before the stress event is not informative nor is it predictive of the observed stress response. Likewise, no relationship was observed between beta diversity and change in cortisol levels.

## Discussion

This research describes the impact of simulated acute real-world stress events on the canine microbiota using a mixed-breed cohort of twenty healthy adult colony dogs, and the exploration to determine whether the microbiota is indicative of a stress response. Faecal samples were collected 24 h before, within 24 h, and 24 to 48 h after each stress occasion to determine changes in the digestive health and gut microbiota composition.

Firstly, to determine whether the stress paradigms had an influence on digestive health, we investigated markers of faecal pH and faecal quality. Faeces score, particularly the extremes of loose or dry, is a key indicator to owners of their pet’s physical and mental health. During times of ill health or stress and anxiety, it is accepted that dysbiosis is related to a change in the faecal output and quality. Faecal pH is often used as a marker of gastrointestinal health and colonisation resistance with an optimal range of 6.0–7.0^24^. Statistically, there were no differences in means for either faecal pH or faecal score throughout the study, and as such there was no observed negative impact on the gastrointestinal health of these dogs. However, the lack of observed differences may well be attributed to our small sample size and the impact this had on statistical power, further exacerbated when sub-setting to stress event types.

There is growing evidence in literature that demonstrates a link between gastrointestinal microbes and the roles they play as part of the gut-brain axis. Changes to the microbiota have been associated with emotional wellbeing, or anxiety-like behaviours and disorders, including depression^8,10^. As such, it is thought that exposure to stress could modify the commensal gut microbiota composition through driving a reduction in health-associated bacteria and increasing some potentially pathogenic species^11^. Across all measures of alpha diversity and beta diversity, we saw no significant impact on the microbiota following the stress events, even after repeated exposure. This may demonstrate that acute and infrequent stressors that occur in the real world may also have no impact on the microbiota. However, further work is needed to determine if factors such as more extreme stressors, an increase in exposure frequency, and/or duration would result in a significant change in the canine gut microbiota.

To explore the taxonomic composition of the pets, we used a canine-specific curated database (DivDB-Canine). This enabled more accurate mapping of the microbiota and greater taxonomic species granularity. We identified species of *Prevotella*, *Phocaeicola*, *Blautia*, and *Faecalibacterium* as some of the most abundant taxa. These have previously been associated with health in dogs across different sequencing platforms^26,27^. These differ with other research conducted to understand the canine gut microbiota and the impact stress, as characterised by “fearful of noises, people, animals” and “always alert”, amongst other criteria, has on dog behaviour. This group identified increases in *Lactobacilli*, *Bifidobacteria*, and Enterobacteriaceae species^13^. However, a decrease in short-chain fatty acid-producing bacteria (i.e., *Blautia*, *Ruminococcus*, and *Faecalibacterium*) has been associated with canine gut dysbiosis, thus resulting in the conclusion that these microbes and their presence are associated with health^28^. We were unable to identify a link between *Blautia* species and canine stress in the literature. However, this species has been associated, in combination with *Lactobacillus* spp., to confer resistance to a negative gastrointestinal response and diarrhoea, which is indicative of ill health, in piglets^29^. A significant increase in abundance of *Blautia* has also been associated with a lower risk of Alzheimer’s Disease in humans (odds ratio, 0.88; 95% CI, 0.79–0.99; *p*-value of 0.028), alongside elevated GABA levels (*p*-value of 0.034)^30^. Further to this, a systematic review of the human gut microbiota revealed species of *Prevotella*, *Blautia,* and *Bifidobacterium* were consistently reduced in patients with autistic spectrum disorder relative to healthy counterparts^31^. Although these are diagnosed neuropsychiatric disorders, it concurs with the presence of *Prevotella* and *Blautia* species in the gut to be associated with positive mental health. This correlates with none of the dogs on the study having been clinically diagnosed with any form of chronic stress or anxiety disorder. It is also important to consider a limitation with comparing across a variety of methods, databases, and pipelines to analyse the canine microbiota is that this may lead to differences in observed results, as demonstrated with recent studies^32,33^.

Relatively few studies have investigated the stress-gut paradigm using routine events in individuals that do not have chronic stress conditions or other neurological indications. Pets are regularly exposed to everyday situations that have the tendency to induce stress. These result in negatively perceived behaviours, such as whining, barking, or inappropriate elimination, that ultimately can harm the pet-owner bond. The cohort participating in this study underwent extensive health checks, including emotional wellbeing and anxiety assessments, and were deemed neurologically and emotionally healthy. Therefore, any changes observed from a behavioural and/or physiological biomarker perspective could be attributed primarily to their response to the stress event paradigms. However, it is important to note that these dogs are not necessarily representative of the general pet dog population and appeared to habituate to the stressors over time. As such, caution should be taken when extrapolating these results to the broader pet dog population.

The cohort of dogs described in this work were enrolled onto two other parallel trials investigating cannabidiol (CBD) and its influence on stress; the findings have since been published^23,34,35^. The dogs in this study were the control/placebo group in these trials and as such did not receive any CBD. However, the overlapping nature of these trials gave us access to previously published data on the physiological and behavioural responses to the stress events. Significant changes in the serum cortisol levels were obtained from dogs when comparing before and after exposure to the stressor car test. This suggests that this paradigm was successful in inducing a stress response in those dogs^23^. Despite a significant physiological response to stress for the car cohort, we were unable to identify a microbial signature, or even certain taxonomic species^23^. Further to this, there was no relationship identified and no correlations could be made for either group. Using this dog cohort, who underwent car travel as a real-world stressor surrogate, it was not possible for the microbiota to be used to predict a canine stress response. Although the sample size when sub-classifying the data to car only could explain the reduced statistical power observed. Regardless of this, we still did not observe any major taxonomic changes.

In addition to the taxonomic composition, we explored the associated functional data. However, analysis of the KEGG KOs did not identify any significant differences across timepoint, irrespective of stress event. Nevertheless, as there is evidence to suggest that the gut-brain axis is likely modulated through changes in metabolites, such as serotonin or dopamine, it is possible that this could occur without observed changes in microbiota composition^5,36^. As such, further exploration into these metabolites may be useful. Future research is also warranted in the wider pet dog population, specifically with those who suffer with chronic stress or a clinical anxiety disorder, as well as more acute exposures to stress-inducing events, to better understand the relationship between canine stress and the gut microbiota.

## Conclusion

Pet dogs will experience a variety of stress and anxiety-inducing events during their lives. These events may be deemed stressful depending on the individual. Such events, often unavoidable, may include car travel and/or separation from their owner, as well as many other routine scenarios. There is a growing interest in understanding how the gut microbiome may modulate, or be modulated, when experiencing stress and anxiety-inducing experiences in a range of mammalian species. Through this work, we have demonstrated that for acute and infrequent stress events, in this study cohort, there is no impact that we could detect with the data we had available on the canine gut microbiota. Furthermore, the pre-existing microbiota was not an indicator of a dog’s response to stress. Additional research is required to investigate the impact that stress has on the canine gut microbiota in a broader dog population and under different stress paradigms.

## Materials and methods

### Animal cohort

The cohort of dogs described in this study were on previously published parallel research trials. The first trial determined the safety of long-term daily feeding of cannabidiol (CBD)^34^. The second examined the anxiolytic effects of a single dose of CBD following exposure to one of two stress-inducing paradigms (car travel or separation)^35^. A third publication explored the anxiolytic effects of daily dosing of CBD on alleviating car travel stress in dogs^23^. The cohort presented in this current study formed the control/placebo group for these trials, and as such did not receive any CBD.

Twenty healthy, adult colony dogs, pair-housed in environmentally enriched kennel facilities at the Waltham Petcare Science Institute (Leicestershire, UK), were recruited to the study. The cohort comprised of 11 neutered males and 9 females (7 neutered and 2 entire), aged between 1.4–7.3 years, from three breeds: 8 Norfolk Terriers, 7 Labrador Retrievers, and 5 Beagles. The dogs experienced their normal daily activities and were exercised consistently throughout the study, according to standard practice for the Institute. All dogs were exposed to the testing environments prior to the study and trained to facilitate sample collections. Dogs were exposed to either car travel (*n* = 10) or were left alone in a room, separated from caregivers (*n* = 10).

### Diet and placebo capsules

Diet was controlled throughout the six-month period. All dogs received standard commercial diet (Royal Canin® Medium Adult Dry) twice daily in addition to a daily Greenies™ dental chew of appropriate size for their breed, as previously published^34^. Nutritional analysis was performed on the main meal diet prior to feeding (Eurofins, UK) to ensure full compliance with the National Research Council^37^. Diet allocation was determined for each individual dog to provide energy levels required for body weight and body condition score maintenance. A consequence of standardising the diet for the entire study population, irrespective of their size, resulted in eight dogs with a lower feed allocation than the target demographic for the diet, i.e., less than 95 kcal/kg^0.75^. As such, supplements of Choline (Choline Chloride; Metabolics®), Selenium (Ionic selenium; Metabolics®) and Riboflavin (Riboflavin 5 Phosphate; Metabolics®) were offered with meals. Dogs were always provided with access to fresh drinking water.

Dogs participating in the study received a placebo capsule within a Royal Canin® Pill Assist pocket with their morning meal. As previously described, food-grade sunflower oil was obtained by Canopy Growth Corporation (Ontario, Canada), and processed by Kazmira LLC (Colorado, USA) into soft gel capsules (bovine origin; RNA Corporation, Illinois, USA)^23,34,35^. To maintain the blinding of the CBD trials, these placebo capsules were manufactured identically. Capsules were analysed by a third-party laboratory to verify the absence of CBD. Within two hours of consumption of the morning meal and placebo capsule, dogs underwent either a car travel or separation test session.

### Habituation to testing scenarios

At the point of enrolment into this study, dogs had undergone some habituation to the testing environments through play and/or training sessions with their handler and to the equipment, as well as experiencing one full test session, as previously published^35^. For car travel, this habituation entailed prior exposure to a metal dog crate, and then voluntarily climbing upon a wooden box or ramp to enter the crate when it was placed in the car’s rear luggage compartment. For the separation event, habituation consisted of regular exposure to the testing room until the dog was considered comfortable and relaxed in the presence of their experienced handler. Dogs were closely monitored throughout the study and at each test session for pre-determined signs of distress and/or compromised welfare based on pre-defined removal criteria.

### Car travel

As previously described by Hunt et al. (2023), each dog participating in the travel paradigm voluntarily entered the rear of the car via a step or a ramp and was secured in a size-appropriate crate^35^. A pre-defined route was driven for 10 min, with the speed not exceeding 10 mph. Dogs were continuously monitored by the driver for the duration of the test through the rear-view mirror for signs of distress. No dogs were removed from the test.

### Separation event

As previously described by Hunt et al. (2023), a purpose-built test room was used for this paradigm^35^. Inside the room where the dogs were left on their own, there was a crate, vet bedding, a water bowl, and other enrichment items including a raised hammock bed, a cardboard box, and some rubber toys. Dogs were led to the room via their handler and then left alone for 45 min. A CCTV system was set up and video footage of the dog in the room was viewed in real time by a researcher in an adjacent room to monitor dog behaviour welfare.

### Serum cortisol

As previously described by Hunt et al. (2023), a small patch of hair was shaved from the dog’s neck and topical anaesthesia (Ethycalm Plus™; Invicta, West Sussex, UK) applied to the area before collection of a 1.2 ml blood sample from the jugular vein^35^. Blood samples were collected into a clot-activating serum tube, kept on ice, and aliquoted and stored at − 20 °C within 60 min of collection. The R&D Systems, Parameter™ cortisol immunoassay (bio-techne, Minneapolis, USA) was used, as per the manufacturer’s instructions with an intra-assay variation of < 3%.

### Faeces sample collection, faecal scoring and pH assessment, and microbiota sample preparation

Freshly produced defecations were collected from dogs at three timepoints: 24 h prior to the stress event (Pre), and after each stress event within 24 h (Post 1), and between 24 to 48 h (Post 2).

Faeces consistency assessments were conducted immediately upon defecation, according to the 17-point Waltham Faeces Scoring System^24^. The pH of the sample was taken before obtaining a 200 mg aliquot from the core of the faeces using sterile disposable spatulas (Fisher Scientific, UK); samples were then stored in Lo-Bind Eppendorf tubes (Eppendorf Ltd., UK) at − 80 °C within 60 min of defecation.

Faecal DNA was extracted using the QIAamp 96 PowerFaecal QIAcube HT Kit (QIAGEN, Germany) with automation of extraction protocols on an epMotion 5075 robot (Eppendorf Ltd., UK). Briefly, suspended faecal material was homogenised by bead beating on a tissue lyser (QIAGEN, Germany) for 5 min at 30 Hz, and treatment with proteinase K (QIAGEN, Germany) for 10 min (all at room temperature). Faecal lysates were applied to the QIAamp 96 well extraction plate under a 900-mbar vacuum prior to washing and elution, according to the manufacturer’s instructions. Purified DNA was analysed by Nanodrop spectrophotometry and quantified with a Qubit 2.0 fluorometer (Invitrogen Europe Ltd., UK), according to manufacturer’s instructions.

### Sequencing and bioinformatic analyses of the gut microbiota

Metagenomics libraries were prepared with a proprietary procedure adapted from the Nextera XT kit (Illumina). Paired-end sequencing (2 × 150bp) was conducted using the Illumina Novaseq platform (Diversigen, USA), according to manufacturer’s instructions, to an average read depth of 7.8 million pairs per library. DNA sequences were filtered for low quality (Q-Score < 30) and length (< 50), and adapter sequences were trimmed using Cutadapt^38^. Host (canine) sequences were removed using Bowtie 2^39^. Post-QC read depth was 7.5 million pairs per library.

Sequencing reads were mapped using fully gapped alignment with BURST^40^ at an identity threshold of 97% to Diversigen’s curated database (DivDB-Canine) containing all bacterial representative genomes in RefSeq with additional manually curated strains. Each sequencing read was assigned to the lowest common ancestor that was consistent across at least 80% of all reference sequences tied for best hit. Average mapping rate (reads mapping / post-QC read depth) was 0.73 at any taxonomic level and 0.52 at species level. Kyoto Encyclopaedia of Genes and Genomes Orthology groups (KEGG KOs)^41^ were observed directly via alignment at an identity threshold of 97% using fully gapped alignment with BURST^40^. Ambiguously mapped reads were excluded from the resulting functional feature table. Based on analysis of mock communities ATCC 1003, low abundant taxa < 0.01% were removed from the dataset to prevent potential noise, as were taxa that were only present in one sample.

### Analysis of alpha diversity in the gut microbiota

The Shannon diversity index and species richness for each sample were calculated on rarefied data to normalise the sequence depths of the samples using R^42^. Each sample was rarefied by taking a random subset of the counts equal to the minimum sequence depth across all samples, and then the Shannon diversity index and species richness for each sample were calculated. Shannon diversity index was calculated using the formula $H=-\sum {p}_{i}ln({p}_{i})$, where ${p}_{i}$ is the proportion for the $i$ th taxa. The species richness was defined as the number of unique taxa found in that sample. This was repeated 50 times by re-sampling, and the mean Shannon diversity index and species richness were calculated for each sample. The Shannon diversity index and species richness of each sample were modelled using a linear mixed effects model separately, with week, sampling occasion, and their interaction as the fixed effect, and individual animal as the random effect to account for multi-level samples using the ‘lme4’ package^43^. Contrasts were made between each of the two Post sampling occasions and the Pre sampling occasion within each week. Family-wise 95% CI and single-stepwise adjusted *p*-values were obtained using the ‘multcomp’ package^44^.

### Analysis of beta diversity in the gut microbiota

Non-metric Multi-dimensional Scaling (nMDS) using Bray-Curtis dissimilarity was conducted on the relative abundances of taxonomic and functional (KEGG KOs) data and visualised using the ‘vegan’ package^45^. Differences between timepoints (Pre, Post 1, and Post 2) were assessed using pair-wise permutational analysis of variance (PERMANOVA) as implemented in the pairwiseAdonis R package^46^ with 999 permutations and constrained by stratification within individual dogs. *P*-values were adjusted using Bonferroni correction.

### Most abundant species in the gut microbiota and differential abundance analysis

The most abundant species were selected based on total abundance over all samples. Taxa not classified at the species level were aggregated in one category labelled “Unknown”. The remaining species were grouped in the “Other” category. A heatmap was created using relative abundances. Differential abundance of all taxa with a prevalence greater than 0.1 was tested with the ‘LinDA’ package^47^. With this method, a linear mixed effects model was fitted on the centred log2-ratio transformed data with “Timepoint Week” as fixed effects and individual dog as random effect. *P*-values were adjusted using the Benjamini-Hochberg method. Alpha was set at 0.05.

### Analysis of faecal quality score, faecal pH, and serum cortisol

Faeces consistency was assessed by trained animal husbandry technicians according to the 17-point Waltham Faeces Scoring System^24^. Faecal quality scores and faecal pH were analysed separately by linear mixed effect model with week, sampling occasion, and their interaction as the fixed effect, and dog as a random effect using the ‘lme4’ package^43^. Normality was assessed using Q-Q plots. Contrasts were made between each of the two Post sampling occasions and the Pre sampling occasion within each week. Family-wise 95% CI and single-stepwise adjusted *p*-values were obtained using the ‘multcomp’ package^44^.

Serum cortisol was analysed as previously described^23^. Briefly, cortisol concentration was fit as the response variable to a linear mixed effect model with timepoint, week, and their interactions as the fixed effects, and dog as the random effect. A log transformation was applied to cortisol concentrations due to visually assessed heteroscedasticity present in the residuals. Estimated means and 95% CI were extracted using the ‘emmeans’ R package and back-transformed^48^. Pair-wise comparisons between timepoints for each week were made with a Tukey adjustment for multiple comparisons. To assess the relationship between serum cortisol changes and microbiota composition changes, we estimated the Pearson correlation between the difference in log-transformed cortisol (Baseline to Post Test) and Bray-Curtis dissimilarity between the Pre and Post samples of the same.

### Ethical approval and consent to participate

This study was approved by the Waltham Animal Welfare and Ethical Review Body (80265) and conducted under the authority of the Animals (Scientific Procedures) Act 1986. All methods were performed in accordance with the relevant guidelines and regulations.

### Supplementary Information

Supplementary Information 1.

Supplementary Information 2.

## Data Availability

The datasets generated during and/or analysed during the current study are available from the corresponding author on reasonable request.

## References

[1] Minamoto, Y., Dhanani, N., Markel, M. E., Steiner, J. M. & Suchodolski, J. S. Prevalence of Clostridium perfringens, Clostridium perfringens enterotoxin and dysbiosis in fecal samples of dogs with diarrhea. *Vet. Microbiol.* **174**, 463–473 (2014). 25458422 10.1016/j.vetmic.2014.10.005

[2] Suchodolski, J. S. *et al.* The fecal microbiome in dogs with acute diarrhea and idiopathic inflammatory bowel disease. *PLoS One* **7**, e51907 (2012). 23300577 10.1371/journal.pone.0051907 PMC3530590

[3] Mayer, E. A. Gut feelings: The emerging biology of gut–brain communication. *Nat. Rev. Neurosci.* **12**, 453–466 (2011). 21750565 10.1038/nrn3071 PMC3845678

[4] Neufeld, K. M., Kang, N., Bienenstock, J. & Foster, J. A. Reduced anxiety-like behavior and central neurochemical change in germ-free mice. *Neurogastroenterol. Motility* **23**, (2011). 10.1111/j.1365-2982.2010.01620.x 21054680

[5] Kubinyi, E., Bel Rhali, S., Sándor, S., Szabó, A. & Felföldi, T. Gut microbiome composition is associated with age and memory performance in pet dogs. *Animals* **10**, 1488 (2020). 32846928 10.3390/ani10091488 PMC7552338

[6] Ambrosini, Y. M. *et al.* The gut-brain axis in neurodegenerative diseases and relevance of the canine model: A review. *Front. Aging Neurosci.* **11**, 461970 (2019). 10.3389/fnagi.2019.00130 PMC6591269 31275138

[7] Galley, J. D. *et al.* Exposure to a social stressor disrupts the community structure of the colonic mucosa-associated microbiota. *BMC Microbiol.* **14**, 1–13 (2014). 10.1186/1471-2180-14-189 PMC4105248 25028050

[8] Liu, R. T. The microbiome as a novel paradigm in studying stress and mental health. *American Psychologist* **72**, 655–667 (2017). 29016169 10.1037/amp0000058 PMC5637404

[9] Bonaz, B., Bazin, T. & Pellissier, S. The vagus nerve at the interface of the microbiota-gut-brain axis. *Front. Neurosci.* **12**, (2018). 10.3389/fnins.2018.00049 PMC5808284 29467611

[10] Foster, J. A., Rinaman, L. & Cryan, J. F. Stress & the gut-brain axis: Regulation by the microbiome. *Neurobiol. Stress* **7**, 124–136 (2017). 29276734 10.1016/j.ynstr.2017.03.001 PMC5736941

[11] Lutgendorff, F., Akkermans, L. & Soderholm, J. The role of microbiota and probiotics in stress-induced gastrointestinal damage. *Curr. Mol. Med.* **8**, 282–298 (2008). 18537636 10.2174/156652408784533779

[12] Sacoor, C., Marugg, J. D., Lima, N. R., Empadinhas, N. & Montezinho, L. Gut-brain axis impact on canine anxiety disorders: new challenges for behavioral veterinary medicine. *Vet. Med. Int.* (2024). 10.1155/2024/2856759 PMC10827376 38292207

[13] Cannas, S. *et al.* Effect of a novel nutraceutical supplement (Relaxigen Pet dog) on the fecal microbiome and stress-related behaviors in dogs: A pilot study. *J. Vet. Behav.* **42**, 37–47 (2021). 10.1016/j.jveb.2020.09.002

[14] Kirchoff, N. S., Udell, M. A. R. & Sharpton, T. J. The gut microbiome correlates with conspecific aggression in a small population of rescued dogs (Canis familiaris). *PeerJ* (2019). 10.7717/peerj.6103 PMC6330041 30643689

[15] Romaniuk, A. C. *et al.* The effect of transportation on puppy welfare from commercial breeding kennels to a distributor. *Animals* **12**, 3379 (2022). 36496902 10.3390/ani12233379 PMC9737031

[16] Radisavljević, K., Vučinić, M., Becskei, Z., Stanojković, A. & Ostović, M. Comparison of stress level indicators in blood of free-roaming dogs after transportation and housing in the new environment. *J. Appl. Anim. Res.* **45**, 52–55 (2015). 10.1080/09712119.2015.1091338

[17] Doring-Schatzl, D. & Erhard, M. H. Undesirable behaviour of dogs in the car-prophylaxis and therapy. *Tierarztliche Praxis Ausgabe Kleintiere Heimtiere* **32**, 170–174 (2004).

[18] Gandia Estellés, M. & Mills, D. S. Signs of travel-related problems in dogs and their response to treatment with dog appeasing pheromone. *Vet. Record.* **159**, 143–148 (2006). 16877680 10.1136/vr.159.5.143

[19] Dinwoodie, I. R., Dwyer, B., Zottola, V., Gleason, D. & Dodman, N. H. Demographics and comorbidity of behavior problems in dogs. *J. Vet. Behav.* **32**, 62–71 (2019). 10.1016/j.jveb.2019.04.007

[20] Feuerbacher, E. N. & Muir, K. L. Using owner return as a reinforcer to operantly treat separation-related problem behavior in dogs. *Animals* **10**, (2020). 10.3390/ani10071110 PMC7401621 32610513

[21] Takeuchi, Y., Houpt, K. A. & Scarlett, J. M. Evaluation of treatments for separation anxiety in dogs. *J. Am. Vet. Med. Assoc.* **217**, 342–345 (2000). 10935036 10.2460/javma.2000.217.342

[22] Lloyd, J. K. F. Minimising stress for patients in the veterinary hospital: Why it is important and what can be done about it. *Vet. Sci.* **4**, (2017). 10.3390/vetsci4020022 PMC5606596 29056681

[23] Flint, H. E., Hunt, A. B. G., Logan, D. W. & King, T. Daily dosing of cannabidiol (CBD) demonstrates a positive effect on measures of stress in dogs during repeated exposure to car travel. *J. Anim. Sci.* **102**, 1–14 (2024). 10.1093/jas/skad414 PMC10810271 38244994

[24] Moxham, G. Waltham feces scoring system—A tool for veterinarians and pet owners: How does your pet rate?. *WALTHAM Focus* **11**, 24–25 (2001).

[25] Khan, I. *et al.* Mechanism of the gut microbiota colonization resistance and enteric pathogen infection. *Front. Cell Infect. Microbiol.* **11**, (2021). 10.3389/fcimb.2021.716299 PMC8733563 35004340

[26] Garcia-Mazcorro, J. F., Dowd, S. E., Poulsen, J., Steiner, J. M. & Suchodolski, J. S. Abundance and short-term temporal variability of fecal microbiota in healthy dogs. *Microbiologyopen* **1**, (2012). 10.1002/mbo3.36 PMC3496977 23170232

[27] Hand, D., Wallis, C., Colyer, A. & Penn, C. W. Pyrosequencing the canine faecal microbiota: Breadth and depth of biodiversity. *PLoS One* **8**, (2013). 10.1371/journal.pone.0053115 PMC3561364 23382835

[28] Pilla, R. & Suchodolski, J. S. The role of the canine gut microbiome and metabolome in health and gastrointestinal disease. *Front. Vet. Sci.* **1**, 498 (2020). 10.3389/fvets.2019.00498 PMC6971114 31993446

[29] Hu, J. *et al.* A microbiota-derived bacteriocin targets the host to confer diarrhea resistance in early-weaned piglets. *Cell Host Microb.* **24**, 817-832.e8 (2018). 10.1016/j.chom.2018.11.006 30543777

[30] Zhuang, Z., Yang, R., Wang, W., Qi, L. & Huang, T. Associations between gut microbiota and Alzheimer’s disease, major depressive disorder, and schizophrenia. *J. Neuroinflam.* **17**, 1–9 (2020). 10.1186/s12974-020-01961-8 PMC7532639 33008395

[31] Liu, F. *et al.* Altered composition and function of intestinal microbiota in autism spectrum disorders: A systematic review. *Transl. Psychiatry* **9**, (2019). 10.1038/s41398-019-0389-6 PMC6351640 30696816

[32] Forry, S. P. *et al.* Variability and bias in microbiome metagenomic sequencing: an interlaboratory study comparing experimental protocols. *Sci. Rep.* **14**, (2024). 10.1038/s41598-024-57981-4 PMC11059151 38684791

[33] Roume, H., Mondot, S., Saliou, A., Le Fresne-Languille, S. & Doré, J. Multicenter evaluation of gut microbiome profiling by next-generation sequencing reveals major biases in partial-length metabarcoding approach. *Sci. Rep.* **13**, (2023). 10.1038/s41598-023-46062-7 PMC10730622 38114587

[34] Bradley, S. *et al.* Long-term daily feeding of cannabidiol is well-tolerated by healthy dogs. *Front. Vet. Sci.* **9**, (2022). 10.3389/fvets.2022.977457 PMC9533147 36213402

[35] Hunt, A. B. G., Flint, H. E., Logan, D. W. & King, T. A single dose of cannabidiol (CBD) positively influences measures of stress in dogs during separation and car travel. *Front. Vet. Sci.* **10**, (2023). 10.3389/fvets.2023.1112604 PMC9992179 36908527

[36] Elfers, K. *et al.* Fecal supernatants from dogs with idiopathic epilepsy activate enteric neurons. *Front. Neurosci.* **18**, (2024). 10.3389/fnins.2024.1281840 PMC10864448 38356649

[37] National Research Council. *Nutrient Requirements of Dogs and Cats* (National Academies Press, 2006).

[38] Martin, M. Cutadapt removes adapter sequences from high-throughput sequencing reads. *EMBnet J.* **17**, 10–12 (2011). 10.14806/ej.17.1.200

[39] Langmead, B. & Salzberg, S. L. Fast gapped-read alignment with Bowtie 2. *Nat. Methods* **9**, 357–359 (2012). 22388286 10.1038/nmeth.1923 PMC3322381

[40] Al-Ghalith, G. & Knights, D. BURST enables mathematically optimal short-read alignment for big data. *bioRxiv* 2020.09.08.287128 (2020).

[41] Kanehisa, M., Furumichi, M., Sato, Y., Kawashima, M. & Ishiguro-Watanabe, M. KEGG for taxonomy-based analysis of pathways and genomes. *Nucleic Acids Res.* **51**, D587–D592 (2023). 36300620 10.1093/nar/gkac963 PMC9825424

[42] R Core Team. R: A language and environment for statistical computing. R Foundation for Statistical Computing. https://www.r-project.org/ (2022).

[43] Bates, D., Mächler, M., Bolker, B. M. & Walker, S. C. Fitting linear mixed-effects models using lme4. *J. Stat. Softw.* **67**, 1–48 (2015). 10.18637/jss.v067.i01

[44] Hothorn, T., Bretz, F. & Westfall, P. Simultaneous inference in general parametric models. *Biom. J.* **50**, 346–363 (2008). 18481363 10.1002/bimj.200810425

[45] Oksanen, J. *et al.* vegan: Community Ecology Package. https://github.com/vegandevs/vegan (2022).

[46] Martinez Arbizu, P. pairwiseAdonis: Pairwise multilevel comparison using adonis. R package version 0.4. (2020).

[47] Zhou, H., He, K., Chen, J. & Zhang, X. LinDA: linear models for differential abundance analysis of microbiome compositional data. *Genome Biol* **23** (2022). 10.1186/s13059-022-02655-5 PMC9012043 35421994

[48] Lenth, R. V. Emmeans: estimated marginal means, aka least-squares means. R package version 1.8.3. https://cran.r-project.org/web/packages/emmeans/emmeans.pdf (2022).

