# Supplementary material for: Impact of acute stress on the canine gut microbiota
Source: Sci Rep. 2024 Aug 14;14:18897. doi: 10.1038/s41598-024-66652-3 (PMC11324789; doi:10.1038/s41598-024-66652-3)
Supplement: Supplementary file 1 — Supplementary Information 1. [file 41598_2024_66652_MOESM1_ESM.docx]

**Supplementary Table 1.** PERMANOVA statistics of functional data. *P*-values below 0.05 are considered statistically significant – denoted with an asterisk (*).

**Supplementary Table 2.** Differential abundance across timepoints in dogs exposed to the car travel stress paradigm. Data represents the three timepoints (Weeks 8, 16, and 24), with samples collected Pre (24 hours prior to the stress event), Post 1 (within 24 hours of the stress event), and Post 2 (between 24 hours to 48 hours after the stress event).

**Supplementary Table 3.** Differential abundance across timepoints in dogs exposed to the separation stress paradigm. Data represents the three timepoints (Weeks 8, 16, and 24), with samples collected Pre (24 hours prior to the stress event), Post 1 (within 24 hours of the stress event), and Post 2 (between 24 hours to 48 hours after the stress event).
